# Prevalence of locomotive syndrome and associated factors in patients receiving hemodialysis

**DOI:** 10.1097/MD.0000000000040007

**Published:** 2025-01-03

**Authors:** Keisuke Hirota, Hiroo Matsuse, Ryuki Hashida, Masato Fukushima, Teturo Imai, Eriko Baba, Hiroshi Tagima, Takuma Hazama, Kei Fukami, Koji Hiraoka

**Affiliations:** a Division of Rehabilitation, Kurume University Hospital, Kurume City, Fukuoka Prefecture, Japan; b Department of Orthopedic Surgery, Kurume University School of Medicine, Kurume City, Fukuoka Prefecture, Japan; c Kurume University Hospital Clinical Engineering Center, Kurume City, Fukuoka Prefecture, Japan; d Division of Nephrology, Department of Medicine, Kurume University School of Medicine, Kurume City, Fukuoka Prefecture, Japan.

**Keywords:** GLFS-25, Kt/V, physical activity, renal rehabilitation

## Abstract

Muscle strength and joint and nervous system functions decline with age and in patients undergoing hemodialysis. The Japanese Orthopaedic Association has defined locomotive syndrome (LoS) as a musculoskeletal disorder primarily caused by aging. Therefore, this study aimed to investigate the prevalence of LoS and identify factors associated with its development in patients undergoing hemodialysis. Patients receiving outpatient hemodialysis at Kurume University Hospital were categorized into LoS and non-LoS groups using the cutoff value of 25-question Geriatric Locomotive Function Scale (GLFS-25). We analyzed differences in malnutrition, biochemical examinations, and Kt/V (a measure of dialysis adequacy) between the 2 groups using Wilcoxon rank-sum tests. Additionally, we evaluated factors that correlated with GLFS-25 through pairwise correlations. Multivariate analysis was performed to determine the independent factors associated with LoS. Nineteen patients were included. The median GLFS-25 score was 18. The LoS group (n = 11) had a significantly higher age (*P* = .0056) and chloride levels than the non-LoS group (n = 8) (*P* = .0175). Furthermore, the Nutritional Risk Index for Japanese Hemodialysis patients, creatinine levels, and Kt/V were significantly lower in the LoS group than in the non-LoS group (*P* = .0156, .0026, and .0163, respectively). The GLFS-25 showed significant correlations with age, total protein levels, C-reactive protein, chloride, creatinine, Nutritional Risk Index for Japanese Hemodialysis patients, and Kt/V (with correlation coefficients of −0.6133, −0.4779, 0.4738, 0.5381, −0.7923, 0.6508, and 0.5747, respectively). Multivariate analysis identified life-space assessment (odds ratio [OR], 3.06; 95% confidence interval [CI], −676 to 674; *P* < .0001) and age (OR, 31.29; 95% CI, −2061 to 2067; *P* = .0007) as risk factors for LoS. Age and physical activity were found to be associated with the development of LoS in patients with end-stage renal disease undergoing outpatient hemodialysis at our hospital. This emphasizes the importance of implementing preventative measures for LoS, especially for older and less physically active patients.

## 1. Introduction

End-stage renal disease (ESRD) is a progressive condition that affects approximately 10% of adults globally.^[1]^ Its continuously increasing prevalence is primarily attributed to population aging.^[1]^ As a result, ESRD has emerged as a significant global public health concern.^[2]^

Chronic diseases have been shown to exacerbate frailty and sarcopenia, and advanced ESRD, particularly end-stage kidney disease, is considered a quintessential condition associated with these issues.^[3]^ Frailty and sarcopenia development in patients with ESRD can be influenced by various factors beyond common risk factors such as insufficient protein intake, aging, and physical inactivity. Factors such as inflammation and diuretic use and other ESRD-specific factors, such as metabolic acidosis and active vitamin D deficiency, also contribute to their development.^[4]^ Studies have revealed that 40% of patients undergoing chronic maintenance dialysis experience both frailty and sarcopenia simultaneously.^[5,6]^ Following the initiation of hemodialysis, patients with ESRD may experience musculoskeletal issues, including a rapid deterioration in physical function.^[7]^ Moreover, frailty and sarcopenia in these patients have been linked to cognitive impairment, fractures, falls, and even reduced survival rates in the context of chronic maintenance hemodialysis.^[5,8,9]^ Thus, frailty in patients with ESRD is clinically significant due to its substantial impact on life expectancy.

The deterioration of musculoskeletal system components, including bones, joints, intervertebral discs, muscles, and nerves, can lead to various symptoms such as pain, limited range of motion, malalignment, balance impairment, and difficulty walking.^[10]^ In 2007, the Japanese Orthopaedic Association introduced the concept of locomotive syndrome (LoS) to identify individuals at high risk of developing gait disorders due to musculoskeletal deterioration.^[10]^ LoS serves as a crucial marker for patients with musculoskeletal disorders who are more likely to require long-term care, thus exerting a significant impact on national social systems.^[11]^ Additionally, the incidence of falls among individuals with LoS is higher than that among community-dwelling older adults.^[11]^ Notably, LoS is associated with the quality of life of community-dwelling older adults^[12]^ and is recognized as a risk factor for older adults who require long-term care due to sarcopenia and frailty.^[13]^ Japan is experiencing the most rapid population aging globally, with patients on dialysis. Consequently, there has been a rise in the proportion of patients undergoing hemodialysis who require walking assistance and long-term care.^[14,15]^

However, the prevalence of LoS in patients receiving hemodialysis remains unclear, as there is limited literature on the factors associated with LoS. Therefore, this study aimed to investigate the prevalence of LoS, and the factors associated with it in patients undergoing chronic maintenance hemodialysis in the outpatient setting.

## 2. Methods

### 2.1. Research design

This study employed a retrospective observational design and included patients with ESRD undergoing outpatient hemodialysis at the Kurume University Hospital Renal Services Center between April and June 2022.

### 2.2. Ethics

The study protocol was formulated and performed in accordance with the guidelines of the Declaration of Helsinki and was approved by the Kurume University Ethics Committee (Approval No.: 22144). A comprehensive study description was published on the homepage of the Kurume University Clinical Research Center to offer patients the opportunity to decline participation or withdraw from the study. All data were anonymized to safeguard personal information. This study was registered with the UMIN Clinical Trials Registry (UMIN000050819).

### 2.3. Participants

**Figure 1.**
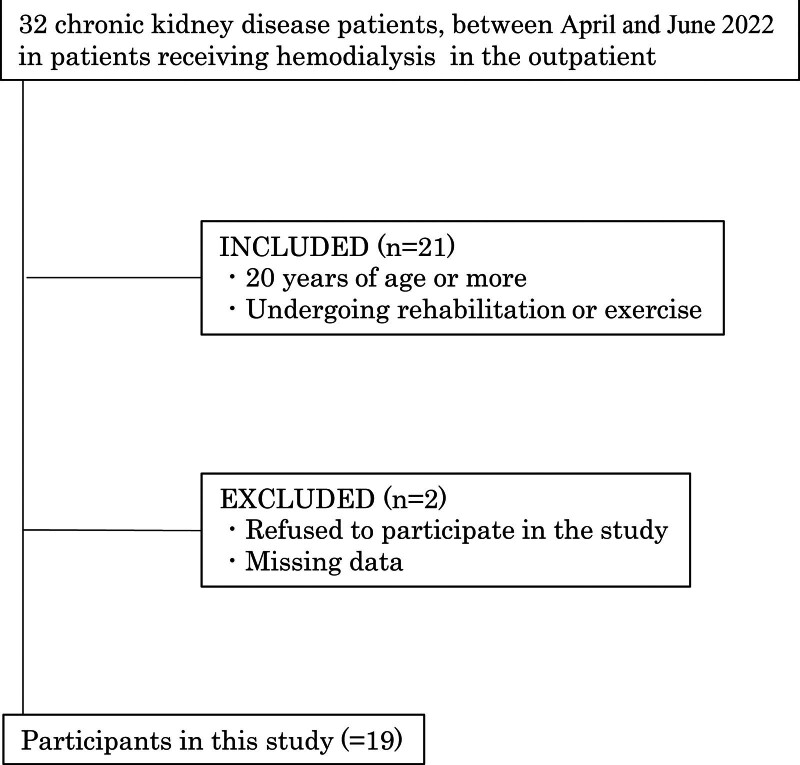
Study flow chart.

The present study included 19 patients with ESRD undergoing chronic maintenance hemodialysis in the outpatient setting at the Kurume University Hospital Renal Services Center between April and June 2022. The inclusion criteria were as follows: patients aged ≥ 20 years and patients receiving rehabilitation therapy. The exclusion criteria were as follows: patients who refused to participate in the study and patients with missing data (Fig. 1).

### 2.4. Assessments and research methods

#### 2.4.1. LoS assessment

LoS was assessed using the 25-item Geriatric Locomotive Function Scale (GLFS-25). The GLFS-25 comprises 25 items, with each item scored on a 5-point scale (0–4), and the final score being the sum of points obtained for each item.^[16]^ The GLFS-25 comprises 4 items related to pain experienced in the past month (items 1–4), 16 items on activities of daily living (ADL) performed in the past month (items 5–15, 17–21), 3 items on social participation (items 16, 22–23), and 2 items on mental health status in the past month (items 24–25). The total score ranges from 0 to 100 points.^[17]^ Participants were classified into either the LoS (score ≥ 16) or the non-LoS group (score < 16) based on previously reported cutoff values.^[12,16]^ The questionnaire interview was conducted by a physical therapist and a nurse.

#### 2.4.2. Assessment of physical activity

Physical activity levels were assessed using the life-space assessment (LSA) questionnaire developed by Baker et al^[18]^ The LSA evaluates the life-space levels, which refers to the spatial range within which an individual engages in activities (categorized as “yes” or “no”), the frequency of engagement (“less than once a week,” “1–3 times a week,” “4–6 times a week,” or “daily”), and the degree of independence during these activities (“with assistance from someone,” “using an assistive device,” or “able to do it alone”). Each category is assigned a weighted score ranging from 1 to 5. The total score on the LSA ranges from 0 to 120 points on a 5-point scale, with higher scores indicating higher levels of physical activity.^[19]^ The LSA was conducted based on the spatial extent, frequency, and degree of independence related to an individual’s physical activity during the past month, using the most recent data from the GLFS-25. The questionnaire interview was conducted by a physical therapist and a nurse responsible for interviewing the participants.

#### 2.4.3. Nutritional status assessment

The Nutritional Risk Index for Japanese Hemodialysis Patients (NRI-JH) is a nutritional screening tool developed using data from hemodialysis patients in Japan. It was derived from cutoff values for body mass index (BMI), albumin, total cholesterol, and creatinine.^[20]^ These assessments were conducted on the most recent data from the GLFS-25 and other measures, including their respective assessment dates.

#### 2.4.4. Skeletal muscle mass assessment

Skeletal muscle mass was assessed using the skeletal muscle index (SMI), derived by measuring the skeletal muscle area (SMA) at the level of the third lumbar vertebra (L3) on abdominal computed tomography (CT) scans and dividing it by the square of the individual’s height.^[21]^ ImageJ Version 1.50 (National Institutes of Health, Bethesda, Maryland) was used for this analysis.^[22]^ Two physical therapists who underwent sufficient training and practice in measurements were responsible for image analysis in this study. The SMI was classified as either low or normal based on previously reported cutoff values.^[23]^

#### 2.4.5. Measurement of the visceral fat area and subcutaneous fat area

Visceral fat area (VFA) and subcutaneous fat area (SFA) were measured at the umbilical level on abdominal CT scans.^[21]^ ImageJ Version 1.50 (National Institutes of Health) was utilized for both the measurement of SMA and the assessments of VFA and SMA.^[22]^

#### 2.4.6. Intramuscular adipose tissue content

The intramuscular adipose content (IMAC) was calculated by dividing the average of 4 CT values for the multifidus muscle, measured at a distance from the major blood vessels at the umbilical level on abdominal CT scans, by the average of four CT values for subcutaneous fat.^[24,25]^ The CT scans were performed with a slice thickness of 5 mm and a tube voltage of 120 kV (p), using an automatic tube current on a Revolution EVO machine (GE Healthcare Japan Corporation, Tokyo, Japan). The CT values were measured using the INFINITT PACS 3.0 (Infinite Technology Corporation, Tokyo, Japan). Image analysis was performed by 2 physical therapists.

#### 2.4.7. Kt/V calculation

The Kt/V value was calculated using dry weight, inter-session weight gain, height, age, sex, blood flow, and urea nitrogen values^[26]^ using pre- and post-dialysis biochemical data. Additionally, the most recent data from the GLFS-25 and other sources were also utilized. The values were automatically computed using the DCS-100NX (Nikkiso Co., Ltd., Tokyo, Japan), which is a multi-purpose dialysis monitoring system. Kt/V served as an index of the dialysis dose and was calculated using the method employed in a statistical survey conducted by the Japanese Society for Dialysis Therapy.^[27]^

#### 2.4.8. Statistical analysis

Data are presented as medians and interquartile ranges. The Wilcoxon rank-sum test was employed to compare the LoS and non-LoS groups. Factors that correlated with the GLFS-25 were evaluated using pairwise correlation. Additionally, a multivariate logistic regression analysis was performed to identify the determinants of LoS. All statistical analyses were performed using JMP Pro 16 (SAS Institute Inc., Cary, North Carolina), with statistical significance set at 0.05.

## 3. Results

### 3.1. Patients’ characteristics

A total of 19 patients were enrolled in the study who were undergoing chronic maintenance hemodialysis in an outpatient setting. The median age of the patients was 65 (49–75) years, with a male:female ratio of 8:11 (57.9% female patients). The median BMI was 21.8 (18.7–25.6) kg/m^2^, and the median score on the functional independence measure (FIM) was 125 (115–126) points, indicating that the majority of patients were capable of independently performing ADL. The median values for SMI, IMAC, VFA, and SFA were 28.7 (25.0–35.3) cm^2^/m^2^, −0.51(−0.59 to 0.46), 46.4 (29.9–101.9) cm^2^, and 71.0 (53.1–129.5) cm^2^, respectively (Table 1). The median values of the biochemical data items were as follows: albumin: 3.5 (3.3–3.8) g/dL, C-reactive protein (CRP): 0.16 (0.05–0.61) mg/dL, total cholesterol: 158 (137–190) mg/dL, triglyceride: 102 (78–157) mg/dL, blood urea nitrogen: 58 (55–67) mg/dL, and creatinine: 10.8 (8.5–11.7) mg/dL. The median scores on the LSA and NRI-JH were 62 (36–110) and 5 (3–11), respectively. Regarding dialysis-related factors, the median weight gain rate was 1.03 (1.02–1.04), the median length of time on dialysis was 15.9 (5.3–22.9) years, and the median Kt/V value was 1.94 (1.46–2.04) (Table 1).

**Table 1:** Univariate analysis based on the presence or absence of LoS

	All patients	LoS group	Non-LoS group	*P* values
	Median (interquartile range)	Median (interquartile range)	Median (interquartile range)	
Number	19	11	8	
Age (years)	65 (49–75)	75 (60–76)	47 (44–63)	0.0056
Sex (female/male)	11/8 (57.9%/52.1%)	7/4 (63.6%/36.4%)	4/4 (50.0%/50.0%)	0.5522
BMI (kg/m^2^)	21.8 (18.7–25.6)	19.2 (17.9–22.9)	24.0 (19.8–27.1)	0.1074
SMI (cm^2^/m^2^)	28.7 (25.0–35.3)	28.3 (25.0–30.3)	35.2 (27.4–17.1)	0.0758
SMI (low/normal)	15/4 (78.9%/21.1%)	10/1 (90.9%/9.1%)	5/3 (62.5%/37.5%)	0.1309
IMAC	−0.51 (−0.59 to 0.46)	−0.51 (−0.85 to 0.45)	−0.52 (−0.54 to 0.49)	0.7726
VFA (cm^2^)	46.4 (29.9–101.9)	37.3 (24.6–86.3)	60.7 (31.9–103.3)	0.4828
SFA (cm^2^)	71.0 (53.1–129.5)	70.8 (43.1–121.3)	96.0 (66.0–211.1)	0.2312
Hemoglobin (g/dL)	11.3 (10.6–11.9)	11.0 (10.7–11.9)	11.4 (10.3–12.3)	0.8685
White blood cells (×10^3^/µL)	6.0 (5.6–7.1)	6.0 (5.6–7.1)	6.4 (5.2–8.7)	0.591
Platelets (×10^3^/mm^3^)	192 (163–272)	209 (151–272)	191 (167–294)	0.7726
Total protein (g/dL)	6.6 (6.0–6.8)	6.7 (6.4–6.8)	6.3 (6.0–6.8)	0.2137
Albumin (g/dL)	3.5 (3.3–3.8)	3.3 (3.1–3.8)	3.6 (3.4–3.8)	0.1802
CRP (mg/dL)	0.16 (0.05–0.61)	0.25 (0.07–0.85)	0.13 (0.04–0.52)	0.2997
Total cholesterol (mg/dL)	158 (137–190)	156 (137–189)	163 (125–186)	0.9671
Triglycerides (mg/dL)	102 (78–157)	112 (59–157)	99 (81–187)	0.4826
Urea nitrogen (mg/dL)	58 (55–67)	57 (55–72)	61 (56–66)	0.9341
Creatinine (mg/dL)	10.8 (8.5–11.7)	9.1 (7.6–11.0)	12.1 (11.0–13.8)	0.0026
Sodium (mmol/L)	139 (137–140)	139 (137–141)	138 (133–140)	0.2114
Potassium (mmol/L)	4.8 (4.3–5.0)	4.8 (4.3–5.0)	4.8 (4.4–5.1)	0.6784
Chloride (mmol/L)	102 (98–104)	104 (102–106)	100 (95–100)	0.0175
SIDE (0/1/2a/2b/3/4)	1/1/4/2/3/8	1/1/3/2/2/2	0/0/1/0/1/6	0.2185
FIM	125 (115–126)	122 (111–125)	126 (125–126)	0.0098
LSA (points)	62 (36–110)	48 (24–62)	115 (95–120)	0.0021
NRI-JH (points)	5 (3–11)	8 (4–11)	3 (0–7)	0.0156
Weight gain rate	1.03 (1.02–1.04)	1.03 (1.02–1.04)	1.03 (1.03–1.05)	0.1236
Length of time on dialysis (years)	15.9 (5.3–22.9)	15.9 (5.3–22.4)	14.7 (5.3–24.1)	0.8365
Kt/V	1.94 (1.46–2.04)	1.59 (1.36–2.02)	2.04 (1.80–2.09)	0.0163

BMI = body mass index, CRP = C-reactive protein, FIM = functional independence measure, IMAC = intramuscular adipose tissue content, LoS = locomotive syndrome, LSA = life-space assessment; NRI-JH = nutritional risk index for Japanese hemodialysis patients; SFA = subcutaneous fat area, SMI = skeletal muscle index, VFA = visceral fat area.

### 3.2. Comparison between the LoS and non-LoS groups

Based on the cutoff values from the GLFS-25, 11 patients (57.9%) were classified into the LoS group, and 8 (42.1%) into the non-LoS group. Differences in basic attributes, body composition, biochemical data, nutritional status, and dialysis-related factors between the 2 groups are presented in Table 1. The LoS group was significantly older than the non-LoS group (*P* = .0056), however, there were no significant intergroup differences in terms of sex, BMI, SMI, VFA, or SFA scores (Table 1). Regarding biochemical data, the creatinine levels were significantly lower in the LoS group (*P* = .0026), while no significant differences were observed in other items of the biochemical data. The FIM and LSA scores were significantly lower in the LoS group than in the non-LoS group (*P* = .0098,.0021). The NRI-JH score was significantly higher in the LoS group (*P* = .0156). In terms of dialysis-related factors, no significant differences were observed in weight gain rate or the length of time on dialysis. However, the Kt/V value was significantly lower in the LoS group than in the non-LoS group (*P* = .0163).

### 3.3. Correlation between the GLFS-25 and each item

The coefficients of correlation between the GLFS-25 scores and body composition, basic attributes, biochemical data, nutritional status, and dialysis-related factors are presented in Table 2. Significantly positive correlations were observed between the GLFS-25 score and age (*P* = .0101), total protein (*P* = .0385), chloride (*P* = .0175), and the NRI-JH score (*P* = .0026) (Table 2). However, significantly negative correlations were observed between the GLFS-25 score and SMI (*P* = .0305), IMAC (*P* = .0175), CRP (*P* = .0404), creatinine (*P* < .001), FIM score (*P* < .001), LSA score (*P* = .0001), and Kt/V (*P* = .0068) (Table 2).

**Table 2:** Correlation with the 25-question geriatric locomotive function scale.

Items	Correlation coefficients	*P* values
Age	0.5747	0.0101
BMI	−0.3589	0.1313
SMI	−0.4968	0.0305
IMAC	−0.5381	0.0175
VFA	−0.2142	0.3785
SFA	−0.281	0.2439
Hemoglobin	0.0662	0.7877
White blood cells	−0.2242	0.3562
Platelets	−0.028	0.9093
Total protein	0.4779	0.0385
Albumin	−0.419	0.0741
CRP	0.4738	0.0404
Total cholesterol	0.0617	0.802
Triglycerides	−0.1474	0.547
Urea nitrogen	0.0894	0.7158
Creatinine	−0.7923	<.001
Sodium	0.282	0.2421
Potassium	−0.2691	0.2653
Chloride	0.5381	0.0175
FIM	−0.7808	<.001
LSA	−0.7716	0.0001
NRI-JH	0.6508	0.0026
Weight gain rate	−0.4472	0.0627
Length of time on dialysis	0.1899	0.4361
Kt/V	−0.6133	0.0068

BMI = body mass index, CRP = C-reactive protein, FIM = functional independence measure, IMAC = intramuscular adipose tissue content, LSA = life-space Assessment; NRI-JH = Nutritional Risk Index for Japanese hemodialysis patients, SFA = subcutaneous fat area; SMI = skeletal muscle index; VFA = visceral fat area.

### 3.4. Multivariate analysis on LoS

A multivariate analysis was performed to investigate the independent determinants of LoS. Through a stepwise logistic regression analysis, we identified advanced age (*P* = .0007) and low LSA score (*P* < .0001) as independent determinants of LoS (Table 3).

**Table 3:** Multivariate analysis on the presence or absence of locomotive syndrome.

Items	Unit	Odds ratios	95% confidence interval	*P* values
Age	1	31.29	−2061 to 2067	.0007
LSA	1	3.06	−676 to 674	<.0001

LSA = life-space assessment.

## 4. Discussion

Our results showed that the prevalence of LoS among patients undergoing chronic maintenance hemodialysis in the outpatient setting at our hospital was 57.9%. Furthermore, we identified age and LSA scores as independent determinants of LoS.

Among the patients receiving chronic maintenance hemodialysis included in this study, 57.9% were found to have LoS. In contrast, a study by Kimura et al^[28]^, which investigated the prevalence of LoS using the GLFS-25 in a nationwide general population of 4500 individuals in their 40s–70s, reported a prevalence of 10.2%,^[28]^ although the participants in the current study were of the same age range. The discrepancy between the previous research and this study lies in the differences between the general population and ESRD patients on chronic maintenance hemodialysis. The incidence of sarcopenia, which shares similarities with LoS, is higher in individuals with ESRD patients on chronic maintenance hemodialysis than in the general population.^[29]^ As ESRD progresses and hemodialysis is initiated, patients experience a decline in cardiovascular and musculoskeletal function. This deterioration may be attributed to factors such as metabolic acidosis, low protein intake, systemic inflammation, and reduced physical activity.^[30]^ Therefore, the prevalence of LoS can be presumed to be higher among patients receiving chronic maintenance hemodialysis than among the general population.

The present study established a correlation between LoS and serum creatinine levels, with patients diagnosed with LoS exhibiting significantly lower serum creatinine levels; although the nature of the association between LoS and serum creatinine levels remains uncertain. Clinically, serum creatinine levels serve as a measure of kidney function,^[31]^ and are commonly used to evaluate the estimated glomerular filtration rate.^[32]^ Additionally, in patients receiving chronic maintenance hemodialysis, serum creatinine levels serve as a biomarker for skeletal muscle metabolism and are correlated with skeletal muscle mass.^[33]^ LoS is a syndrome characterized by the functional impairment of the musculoskeletal system, including skeletal muscles, and is thought to reflect skeletal muscle loss. Therefore, serum creatinine levels may prove useful in assessing LoS in patients undergoing chronic maintenance hemodialysis.

Furthermore, the results of this study indicate a possible link between LoS and Kt/V. Tentori et al^[34]^ reported that among patients receiving chronic maintenance hemodialysis, those who engaged in regular exercise had significantly higher Kt/V values. A study on factors associated with physical function in patients receiving maintenance hemodialysis on an outpatient basis found that walking speed, stair ascent/descent time, and timed chair stands were all significantly associated with Kt/V,^[9]^ indicating that Kt/V is associated with physical function. Additionally, in a cohort study on frailty and mortality in older adults receiving hemodialysis, Guo et al^[35]^ established that Kt/V was a determinant for frailty. Furthermore, in a randomized controlled trial, Dong et al^[36]^ found that exercise therapy improved physical function and led to a significant increase in Kt/V in patients receiving chronic maintenance hemodialysis.^[36]^ As mentioned previously, the calculation of Kt/V takes into account factors such as dry weight, inter-session weight gain, height, age, sex, blood flow, and urea nitrogen levels. Hasan et al^[37]^ reported that the average dry weight of patients with a higher Kt/V was higher than that of patients with a lower Kt/V. Thus, higher Kt/V may be associated with improved dialysis adequacy, leading to decreased gastrointestinal tract problems, improved appetite, reduced inflammation, and improved anabolism.^[37,38]^ Furthermore, physicians may increase the blood flow rate and dialyzer surface area as a result of improved physical conditions. Thus, Kt/V is associated with exercise habits, physical function, frailty, and the implementation of exercise therapy and LoS may be a factor associated with the efficiency of hemodialysis.

In this study, we established that age is a determinant for LoS. The prevalence of skeletal muscle loss due to LoS is widespread among the elderly, which progresses with age.^[39]^ Notably, approximately 15% of the adult population in the United States suffers from ESRD, according to the Centers for Disease Control and Prevention. The incidence of ESRD is 12% for individuals aged 45–64 years, 6% for those aged 18–44 years, and 38% for those aged ≥ 65 years.^[40]^ Thus, a strong correlation exists between ESRD and the aging process; ESRD exacerbates the effects of aging while aging accelerates the progression of ESRD. Furthermore, frailty has been reported to negatively impact both the kidneys and the body as a whole.^[41]^ Moreover, abnormalities in the musculoskeletal system and ESRD are intimately linked with aging and are believed to mutually influence one another.

Our findings suggest that LSA may be a risk factor for LoS, as the level of physical activity provides important prognostic information regarding mortality and the incidence of functional impairment in community-dwelling older adults.^[42]^ A reduction in physical activity levels can exacerbate conditions such as cardiovascular disease, neoplastic diseases, cognitive decline, and noncommunicable diseases.^[43]^ Therefore, it is common for individuals with ESRD, including those on dialysis, to exhibit decreased levels of physical activity.^[44]^ Moreover, the majority of patients with ESRD do not meet the recommended physical activity guidelines, which include engaging in moderate activity at least 5 times per week or vigorous activity at least 3 times per week.^[45]^ Thus, decreased physical activity in individuals with ESRD is indicative of physical frailty. Studies suggest that patients with advanced ESRD exhibit low levels of activity and significant skeletal muscle atrophy before the initiation of dialysis.^[46]^ Furthermore, patients receiving chronic maintenance hemodialysis may experience difficulty with basic ADL and mobility tasks, such as ascending/descending stairs, even if they can walk independently.^[47]^ A scoping review by Lambert et al^[48]^ reported that decreased physical activity in patients receiving chronic maintenance hemodialysis is associated with reduced quality of life, diminished physical function, increased physical pain, longer hospital stays, and decreased overall survival. These findings suggest that reduced physical function in patients receiving maintenance hemodialysis can lead to decreased physical activity and impaired mobility, which can contribute to LoS.

Nonetheless, the present study had several limitations. As this was a single-center study, the number of eligible patients was limited, and the potential for subject selection bias could be ruled out. Furthermore, as this was a retrospective study, physical function assessments such as the 2-step and stand-up tests were not performed. Therefore, a prospective, multicenter validation study that takes these factors into account may provide further insight into LoS in ESRD patients undergoing outpatient hemodialysis.

In this study, the prevalence of LoS in patients undergoing chronic maintenance hemodialysis in the outpatient setting at our hospital was found to be 57.9%. Age and LSA score were identified as possible determinants of LoS, highlighting the importance of proactive LoS prevention measures for older adults and physically inactive individuals with ESRD. Thus, in the future, we aim to contribute to the development of renal rehabilitation medicine by further evaluating LoS and physical function in ESRD patients undergoing chronic maintenance dialysis.

## Acknowledgments

We are grateful to the doctor and nurse staff of the Dialysis Unit for their dedication to the care and protection of patients on hemodialysis treatment. We would like to thank Editage (www.editage.jp) for English language editing.

## Author contributions

**Conceptualization:** Keisuke Hirota, Hiroo Matsuse, Ryuki Hashida, Takuma Hazama.

**Data curation:** Keisuke Hirota, Hiroo Matsuse, Masato Fukushima, Teturo Imai, Eriko Baba, Hiroshi Tagima, Takuma Hazama.

**Formal analysis:** Keisuke Hirota, Ryuki Hashida, Teturo Imai, Hiroshi Tagima.

**Investigation:** Keisuke Hirota, Hiroo Matsuse, Ryuki Hashida, Teturo Imai, Eriko Baba, Hiroshi Tagima.

**Methodology:** Keisuke Hirota, Ryuki Hashida.

**Project administration:** Keisuke Hirota, Hiroo Matsuse.

**Resources:** Keisuke Hirota.

**Software:** Keisuke Hirota.

**Validation:** Keisuke Hirota.

**Writing—original draft:** Keisuke Hirota, Ryuki Hashida, Masato Fukushima, Teturo Imai, Eriko Baba, Hiroshi Tagima.

**Funding acquisition:** Hiroo Matsuse, Kei Fukami, Koji Hiraoka.

**Supervision:** Hiroo Matsuse.

**Writing—review & editing:** Hiroo Matsuse, Kei Fukami, Koji Hiraoka.

**Visualization:** Masato Fukushima, Teturo Imai.
